# The complete mitochondrial genome of *Hexagrammos agrammus* (Scorpaeniformes: Hexagrammidae) by next-generation sequencing

**DOI:** 10.1080/23802359.2020.1780971

**Published:** 2020-06-17

**Authors:** Dongping Ji, Jun Liang, Pengfei Li, Tianxiang Gao, Shengyong Xu

**Affiliations:** a Agricultural Machinery Service Center of Fangchenggang, Fangchenggang, Guangxi, China; b Key Laboratory of Sustainable Utilization of Technology Research for Fishery Resource of Zhejiang Province, Zhoushan, Zhejiang, China; c Fishery College, Zhejiang Ocean University, Zhoushan, Zhejiang, P.R. China

**Keywords:** Hexagrammidae, mitogenome, *Hexagrammos agrammus*, next-generation sequencing

## Abstract

The complete mitochondrial genome of the *Hexagrammos agrammus* is presented in this study. The mitochondrial genome is 16,512 bp long and consists of 13 protein-coding genes, 2 rRNA genes, 22 tRNA genes, and a control region. The gene order and composition were similar to those of most other vertebrates. The nucleotide compositions of the heavy strand are 17.27% of G, 26.10% of T, 26.85% of A, and 29.78% of C. With the exception of the NADH dehydrogenase subunit 6 (ND6) and 8 tRNA genes, all other mitochondrial genes are encoded on the heavy strand. The phylogenetic analysis by neighbour-joining (NJ) method showed that *H. agrammus* has the closer relationship with *Hexagrammos otakii* and *Hexagrammos lagocephalus* in the phylogenetic relationship.

Spotty belly greenling, *Hexagrammos agrammus*, is an endemic species in the northwestern Pacific, being locally distributed along the coasts of the China. This species is commercially important in the northwestern Pacific and also caught by game fishing. Several studies have been carried out regarding the morphology, systematic and ecology of the species (Kanamoto 1979; Crow et al. 2004; Kwak et al. 2005; Kimura et al. 2007). However, studies on the genetic diversity of *H. agrammus* have little been conducted yet. Assessments of genetic information are essential to develop strategies for the conservation and management of fisheries resources. The next-generation sequencing (NGS) technologies, such as Illumina, allow considerable numbers of sequence data to be rapidly and efficiently characterized, which make it particularly feasible for mitogenomes (Gilbert et al. 2007). Moreover, Illumina sequencing has been successfully used to assemble the mitogenomes of fish species (Cui et al. 2009). Therefore, we determined to sequence the complete mitochondrial genome of *H. agrammus* using the next-generation sequencing (NGS) techniques strategy in order to find new DNA markers for the studies on genetics of *H. agrammus*.

The specimen of *H. agrammus* was collected from the coastal water of Qingdao (36.41°N, 120.77°E), China, during May 2019. The examined specimen was preserved at Fisheries Ecology and Biodiversity Laboratory in Zhejiang Ocean University under specimen accession NO. ZJOU-04057 and the muscle tissue was preserved in 95% ethanol. The genomic DNA was extracted from dorsal-lateral muscles (30 mg) using Rapid Animal Genomic DNA Isolation Kit (Sangon Biotech Co., Ltd., Shanghai, CN). A genomic library was established and followed by next-generation sequencing. Whole genome resequencing (sequencing depth 50X) was conducted by using Illumina Hiseq4000 platform with the sequencing insertion of 350-bp. Quality check for sequencing data was done by FastQC (Andrews 2010) and the filtered clean data were assembled and mapped to complete mitogenome sequence using NOVOPlasty v3.7.2 (Dierckxsens et al. 2017). The assembled sequence was subsequently annotated using the online Mitochondrial Genome Database of Fish server (Iwasaki et al. 2013) and the MITOS Web Server (Bernt et al. 2013).

The final sequence has been deposited in GenBank with accession number MT363637. The complete mitochondrial genome of *H. agrammus* (16,512 bp in length) consists of 13 protein-coding genes, 22 transfer RNA genes (tRNA), 2 ribosomal RNA genes (12S rRNA and 16S rRNA), and 2 non-coding control regions (control region and origin of light-strand replication). The arrangement of all genes is identical to that of most vertebrates (Wang et al. 2008; Chen 2013; Chiang et al. 2013). Most of the genes are encoded on the heavy strand (H-strand), except for the eight tRNA genes (-Gln, -Ala, -Asn, -Cys,-Tyr, -Ser, -Glu and -Pro) and one protein-coding gene (NADH dehydrogenase subunit 6, ND6). The overall nucleotide compositions of the heavy strand in descending order are 17.27% of G, 26.10% of T, 26.85% of A, and 29.78% of C, with a slight A + T-rich feature (54.95%). All the protein-coding genes begin with an ATG start codon except for COI started with GTG. Three types of stop codons revealed are TAA (COI, ATP8, ATP6, COIII, ND4L, ND5), TAG (ND1, ND2, ND3, ND6), and T (COII, ND4, Cytb). These features are common among vertebrate mitochondrial genome, and TAA is supposed to be appeared via posttranscriptional polyadenylation (Ojala et al. 1981). The longest one is ND5 gene (1839 bp) among protein-coding genes, whereas the shortest is ATPase 8 gene (168 bp). The two ribosomal RNA genes, 12S rRNA (947 bp), and 16S rRNA (1,692 bp), are located between tRNA-Phe (GAA) and tRNA-Leu (TAA), and are separated by the tRNA-Val gene with the same situation found in other vertebrates. Most genes are either abutted or overlapped. The 22 tRNA genes vary from 66 to 74 bp in length. All these could be folded into the typical cloverleaf secondary structure although numerous non-complementary and T–G base pairs exist in the stem regions. The control region was 839 bp in length, located between tRNA-Pro (TGG) and tRNA-Phe (GAA) gene. The nucleotide composition of control region was 32.65% of A, 20.26% of C, 16.45% of G, 30.63% of T.

To confirm the phylogenetic position of *H. agrammus* among family Hexagrammidae, a neighbour-joining (NJ) tree was reconstructed using MEGA6 (Tamura et al. 2013), with the complete mtDNA sequences from 7 species of family Hexagrammidae. As shown in Figure 1, the *H. agrammus* has a relatively closer relationship with *Hexagrammos otakii* and *Hexagrammos lagocephalus*. Furthermore, the species among *Hexagrammos* and *Pleurogrammus* clustered into a monophyletic clade suggested the closer relationship between them than between *Ophiodon elongatus*. The information of the mitogenome will be beneficial for future phylogenetic studies and specimen identification of Hexagrammidae species.

**Figure 1.**
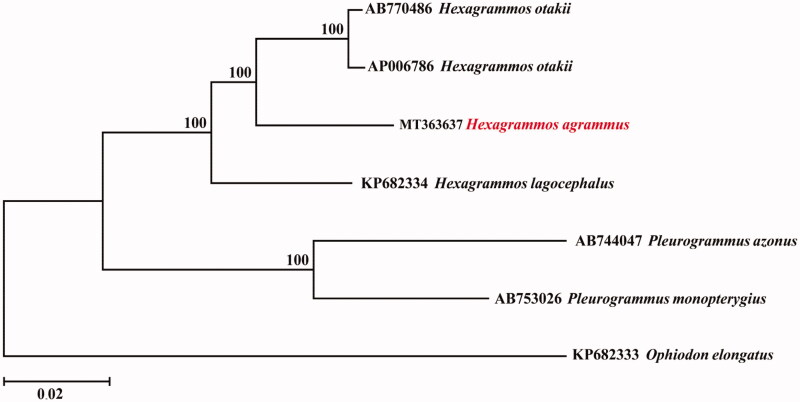
Phylogenetic relationships (neighbor-joining tree) for 7 species of family Hexagrammidae. Numbers on each node are bootstrap values of 1000 replicates.

## Data Availability

The data that support the findings of this study is openly available in GenBank of NCBI at https://www.ncbi.nlm.nih.gov under accession number MT363637.
